# Mapping the Multiple Graded Contributions of the Anterior Temporal Lobe Representational Hub to Abstract and Social Concepts: Evidence from Distortion-corrected fMRI

**DOI:** 10.1093/cercor/bhw260

**Published:** 2016-10-17

**Authors:** Richard J. Binney, Paul Hoffman, Matthew A. Lambon Ralph

**Affiliations:** 1 Neuroscience and Aphasia Research Unit (NARU), School of Psychological Sciences, University of Manchester, Manchester M13 9PL, UK; 2 Eleanor M. Saffran Center for Cognitive Neuroscience, Department of Communication Sciences and Disorders, Temple University, Philadelphia, PA 19122, USA; 3 Center for Cognitive Ageing and Cognitive Epidemiology, Department of Psychology, University of Edinburgh, EH8 9JZ, UK

**Keywords:** anterior temporal lobe, conceptual knowledge, fMRI, semantic memory, social cognition

## Abstract

A growing body of recent convergent evidence indicates that the anterior temporal lobe (ATL) has connectivity-derived graded differences in semantic function: the ventrolateral region appears to be the transmodal, omni-category center-point of the hub whilst secondary contributions come from the peripheries of the hub in a manner that reflects their differential connectivity to different input/output modalities. One of the key challenges for this neurocognitive theory is how different types of concept, especially those with less reliance upon external sensory experience (such as abstract and social concepts), are coded across the graded ATL hub. We were able to answer this key question by using distortion-corrected fMRI to detect functional activations across the entire ATL region and thus to map the neural basis of social and psycholinguistically-matched abstract concepts. Both types of concept engaged a core left-hemisphere semantic network, including the ventrolateral ATL, prefrontal regions and posterior MTG. Additionally, we replicated previous findings of weaker differential activation of the superior and polar ATL for the processing of social stimuli, in addition to the stronger, omni-category activation observed in the vATL. These results are compatible with the view of the ATL as a graded transmodal substrate for the representation of coherent concepts.

## Introduction

Through a process of transmodal distillation of verbal and non-verbal experience, conceptual knowledge brings meaning to objects, people and words, and is the foundation for sophisticated and flexible interaction with our environment and other agents within it. A prominent view regarding the neural organization of conceptual knowledge is that the bilateral anterior temporal lobe (ATL) constitutes a central representational substrate which, alongside distributed modality-specific “spoke” areas, contributes to coherent concepts (Rogers et al. 2004; Lambon Ralph et al. 2010; Lambon Ralph 2014). The genesis of this hypothesis was neuropsychological investigation of patients with semantic dementia (SD; a variant of frontotemporal dementia) whose ATL-centered atrophy leads to selective and gradual dissolution of conceptual knowledge, encompassing both verbal and non-verbal domains (Bozeat et al. 2000; Hodges and Patterson 2007). It has been bolstered and extended by a recent accumulation of convergent evidence from functional imaging (including PET, fMRI and MEG), intracranial recording studies and transcranial magnetic stimulation studies of healthy subjects (Vandenberghe et al. 1996; Marinkovic et al. 2003; Pobric et al. 2007; Lambon Ralph et al. 2009; Binney et al. 2010; Mion et al. 2010; Chan et al. 2011; Visser and Lambon Ralph 2011; Guo et al. 2013; Abel et al. 2014, 2016; Shimotake et al. 2014). Under this view, the bilateral ATL is considered crucial for the representation of all types of concepts, including knowledge of concrete objects and more abstract conceptual constructs (Jefferies et al. 2009; Pobric et al. 2009).

These recent, multi-method studies have led to an updated version of the theory which we have termed the “graded” hub-and-spoke model (Binney et al. 2012; Rice et al. 2015a). As predicted by the original hypothesis, both white-matter and functional connectivity studies show that there is strong, multimodal convergence of widespread modality-specific information sources into the anterior temporal lobe (Moran et al. 1987; Binney et al. 2012; Pascual et al. 2013; Jackson et al. 2016). In addition, these investigations have found that the convergence is graded such that some peripheral ATL regions have stronger connectivity to one modality more than the others. For example, STG regions are more strongly connected to auditory and motor areas, polar regions to orbitofrontal cortex and pars orbitalis of the frontal operculum, whilst the ventrolateral ATL is strongly and evenly connected. Distortion-corrected fMRI and intracranial recording studies (using ECoG and grid stimulation) have found that function follows connectivity such that the ventrolateral ATL region appears to be a “hotspot” or center-point of the ATL hub, with strong transmodal and omni-category responses (Binney et al. 2010; Chan et al. 2011; Visser and Lambon Ralph 2011; Visser et al. 2012; Shimotake et al. 2014; Abel et al. 2015). Likewise, representational similarity analysis of fMRI and ECoG data has found evidence of semantic coding and fusing of modality-specific information sources into this region (Peelen and Caramazza 2012; Coutanche and Thompson-Schill 2015; Chen et al. 2016). Around this center-point, the peripheral hub areas tend to generate lower activations, which are somewhat more selective in nature. These variations seem to reflect the graded connectivity profiles. For example, in comparing semantic decisions to pictures, spoken names and environmental sounds, Visser and Lambon Ralph (2011) observed a strong modality-general semantic response in the ventrolateral ATL and an auditory-related semantic response in the anterior STS. A key question for our study was the following: what contribution do these ATL subregions make to the representation of different categories of knowledge? The logic of the graded ATL hub hypothesis would lead to the expectation that the “core” ventrolateral area underpins all types of concept with the additional contribution of other ATL subregions reflecting the connectivity of each area to any critical modalities of information (Hoffman et al. 2015a).

In the present investigation, we explored the representation of social and matched (non-social) abstract words across the graded ATL hub. We picked these semantic domains for several reasons. First, abstract/social concepts and the words we use to describe them, such as “generous”, occur frequently in our daily experiences and are important for guiding meaningful interpersonal behaviors. Secondly, both types of concepts are somewhat less related to external sensory experience than more commonly investigated concrete concepts and may call more upon “internal” affective information (Kousta et al. 2011; Vigliocco et al. 2014). Third, both classical and contemporary studies have linked anterior temporal lobe structures (alongside regions traditionally associated with “social” processing such as the medial prefrontal cortex) with social-affective behavior (Olson et al. 2007; Zahn et al. 2007; Simmons and Martin 2009) including the attribution of mental states (also known as “Theory of Mind”; Frith and Frith 2003), moral cognition (Moll et al. 2005) and processing of affect (Wicker et al. 2003).

Although ATL regions have been generally implicated in social cognition, many key questions remain, particularly in terms of the relationship between social versus general semantic processing. A good example of this puzzle is represented by the classic comparative neurological studies of bilateral, full-depth ATL resection in non-human primates and one human single case-study (Brown and Schafer 1888; Klüver and Bucy 1939; Terzian and Ore 1955; Kling et al. 1993). In the contemporary literature, these investigations are most commonly cited for the post-operative changes in social behavior. The original studies, however, were primarily focussed on establishing that bilateral ATL lesions led to associative as opposed to apperceptive agnosia. Indeed, Klüver and Bucy noted that the primates not only failed to generate the meaning of visual stimuli but they also did not understand familiar auditory stimuli. Such multimodal semantic impairments are, of course, reminiscent of those observed in semantic dementia and, intriguingly, Klüver and Bucy finished their seminal paper with the comment that the primates’ symptom complex was very similar to that described by Arnold Pick in some human patients (for what would later come to be called frontotemporal dementia). Further, in addition to their generalized semantic impairment, personality changes and deficits in socioemotional processing are also features of semantic dementia further implicating the ATL in both the social and general semantic cognitive domains (Thompson et al. 2003; Chan et al. 2009; Binney et al. 2016). On the other hand, in the initial stages of behavioral-variant frontotemporal dementia, atrophy typically extends from orbitofrontal regions to the temporal pole alone (rather than the entire ATL region) without generating the same degree of semantic impairment observed in SD patients (Perry and Hodges 2000). This suggests that socio-affective processing in the ATL could be somewhat localized to agranular/dysgranular polar cortex while damage to posteriorly-adjacent granular ATL neocortex is necessary for general semantic impairments.

The present study used fMRI to evaluate three hypotheses regarding the role of the ATL in social and general semantic processing. First, ATL function could be limited to either to social or general semantic processing. We tested this hypothesis not only by probing social and non-social concepts in the same participant group but also by matching the two sets very closely on key psycholinguistic factors (frequency, imageability and semantic diversity – a measure of how much the meaning of a word shifts over contexts; Hoffman et al. 2013). If the ATL is dedicated to social processing and general semantic representations are generated elsewhere (Simmons and Martin 2009; Skipper et al. 2011), then only social concepts should activate ATL regions. Conversely, if social meanings are just one subtype of concept that a generalized ATL semantic system processes, then no significant differences would be found. The second possibility is that, within the broader ATL region, there are distinct modules that process social and non-social concepts, entirely separately. As such, the two carefully-matched sets of concepts should generate entirely distinct “binary” activation patterns with activation only for the social concepts in one area and the reverse in an alternative ATL region. Finally, according to the connectivity-driven graded ATL hub hypothesis, both types of concept should activate the ventrolateral center-point of the hub whilst a *partial* difference in favor of social concepts might emerge in more superior/polar ATL regions where input from medial limbic temporal and orbitofrontal regions is maximal (through the uncinate fasciculus; Moran et al. 1987; Binney et al. 2012; Pascual et al. 2013; Von Der Heide et al. 2013; Bajada et al. 2016).

For an appropriate evaluation of these alternative hypotheses, it is essential to ensure complete coverage of the ATL with fMRI. Previous investigations have established a number of key factors that influence the likelihood of observing activation in ventral ATL regions (Visser et al. 2010b). One is to ensure a field-of-view sufficiently deep to cover and sample all ATL regions, including the pole and the ventral surface. Second, the sensitivity of conventional gradient-echo functional MRI is not homogenous across the ATL; whilst signal is relatively good in dorsolateral regions, it is particularly poor in inferolateral and ventral regions (Devlin et al. 2000). It is only with the use of techniques that minimize these issues that it has been possible to reliably demonstrate that semantic tasks evoke vATL activation with fMRI (Binney et al. 2010; Visser et al. 2010a; Visser and Lambon Ralph 2011; Visser et al. 2012; Robson et al. 2014; Binney and Lambon Ralph 2015; Jackson et al. 2016). To date, these techniques have not, however, been employed in studies examining the neural correlates of social conceptual processing. Therefore, we also sought to evaluate the potential impact of these methodological factors on prior observations by including in our experimental design a replication of Zahn et al. (2007), a landmark study that probed the ATL contribution to social concepts. Zahn et al. contrasted comprehension of social concepts (e.g., matching *tactless* with *impolite*) with non-social concepts that referred to animal functions (e.g., matching *nutritious* with *useful*). Social concepts selectively activated the superior ATL but no effects were observed in ventral ATL, perhaps due to these methodological factors (also see Ross and Olsen 2010). We investigated vATL activity to Zahn et al.’s stimuli when using an fMRI protocol for obtaining signal in this key semantic region.

## Materials and Methods

### Design Considerations

The design of the present study was focused around two major components. First, was the replication of the Zahn et al. (2007) study, using the original social and non-social (animal-function) stimuli, but utilizing a neuroimaging protocol suitable for obtaining signal from the whole of the ATL including the ventrolateral ATL (vATL). Replication of the Zahn et al findings is important in that it would negate claims that any additional observations (e.g., in the vATL) reflect non-specific differences in the imaging sequences and procedures, or any other aspect of the study design, rather than being of theoretical significance. The second core aim of the study was to contrast social versus non-social concept processing while controlling for differences in three psycholinguistic properties of the stimuli. Zahn and colleagues matched their conditions for lexical frequency and adjusted for differences in imageability using a post hoc regression (rather than matching stimuli for this key variable in advance). Semantic diversity or similar measures were not accounted for. Accordingly, we contrasted their social concept stimuli with a new set of non-social stimuli matched on all three variables, a priori. This approach enables more stringent control of the extraneous variables and reduces the risk of Type I errors that could occur in post hoc regression approaches as a result of over-fitting the data and inadvertently modeling noise.

In addition to differential activation, the present study was equally concerned with regions commonly involved in processing concepts of both types. Within direct contrasts, the logic of the categorical cognitive subtraction approach dictates that common activation will be removed from the resultant statistical maps. To assess common activation, each condition should be contrasted independently with a “baseline” map of activation. Moreover, this should be supplemented with conjunction analyses (Nichols et al. 2005). One approach, as undertaken by Zahn and colleagues, is to contrast against a low-level “rest” condition or passive viewing of a fixation cross. However, we have previously demonstrated that this approach limits sensitivity to detect semantic activation (Visser et al. 2010b), possibly because ongoing processes during rest are themselves semantic (e.g., “idle thought” or internal speech) and therefore subtraction analyses will remove semantic areas (Binder et al. 1999; Humphreys et al. 2015). A more sensitive approach is to use a high-level “active” baseline condition that is equivalent to the experimental conditions in terms of general task requirements but with a minimal semantic component (Binder et al. 1999, 2009; Visser et al. 2010a). In the present study, we used a numerical judgment task that we have previously employed successfully as a suitable, difficulty-matched baseline in previous fMRI semantic investigations (Binney et al. 2010; Hoffman et al. 2015a), as well as in TMS, cortical grid electrode and neuropsychological studies (Pobric et al. 2007; Lambon Ralph et al. 2012; Shimotake et al. 2014).

### Participants

Nineteen healthy, native English-speaking individuals (10 males; age range = 19–39 years, mean age = 25.9, SD = 5.8) took part in the experiment. All had normal or corrected-to-normal vision and all but one were right-handed. None of the subjects had a history of neurological or psychiatric disorders. The experiments were reviewed and approved by the local ethics board.

### Experimental and Baseline Tasks

Participants completed a two-alternative forced-choice (2AFC) semantic decision task. On each trial of the semantic task, participants were presented with a written probe word with two choices below it (the semantically-related target and an unrelated foil). They were asked to select the word that was most similar in meaning to the probe. In the baseline number judgment trials, participants were presented with a probe number between 1 and 99, along with two numerical choices. They were instructed to select the number closest in value to the probe. Previous studies have found that this task was similar in difficulty (in terms of response time) to semantic judgments (Pobric et al. 2007; Hoffman et al. 2010). Therefore, the baseline task required similar levels of attention and general cognitive effort, but minimal semantic processing.

### Stimuli

Twenty four of the 25 positively-valenced social concept triads from the Zahn et al. (2007) study were used as well as the 24 negatively-valenced social concept triads and the 24 animal function concept triads (we are grateful to Roland Zahn for making these stimuli available to us; for a previous description, see Zahn et al. 2007, 2009) . Given that (a) Zahn and colleagues (Zahn et al. 2007, 2009) found that ATL activation was independent of valence, and that (b) we were only concerned with the social versus general concept distinction per se, the negatively and positively valenced trails were treated as a single condition with 48 trials. To ensure equal sampling of conditions, each of the 24 animal concept triads was repeated once, with the position of the target and foil reversed (maintaining a 50% probability of the target appearing in the left or right position, as in all conditions), providing a total of 48 trials in this condition.

We also generated a new, additional semantic condition with 48 trials, comprising of non-social stimuli matched to the social concept stimuli on imageability, semantic diversity and log lexical frequency, hereafter referred to as “matched-abstract concepts.” Probe words were selected from a list for which semantic diversity had been calculated by Hoffman et al. (2011, 2013). Lexical frequencies were obtained from the CELEX database (Baayen et al. 1993) and imageability ratings were obtained from the MRC database (Coltheart 1981). Care was taken to exclude words that describe or can be directly associated with emotional affect (e.g., “misery”), personality traits (e.g. “selfish”) or explicitly relate to interpersonal interaction (e.g., “blame” or “fight”) and therefore could be considered socially relevant. The semantically-related target and the unrelated foil (choice words) were also selected with efforts to match their mean psycholinguistic variables to that of the probe words. Examples of the stimuli are given in Table 1.

**Table 1:** Example stimuli

	Probe	Target	Distractor
Social concepts		
	BRIGHT	SMART	TRUTHFUL
	AMBITIOUS	EAGER	LIVELY
	CAREFUL	CAUTIOUS	HONESTY
Animal function concepts		
	TRAINABLE	RIDDEN	POISONOUS
	SWIMS	FLOATS	FLOCK
	FAST	FLIES	BURROWS
Matched-abstract concepts		
	EDITION	VERSION	PATENT
	VIVID	INTENSE	DYNAMIC
	BLEND	MERGE	TOIL

Pilot behavioral testing was undertaken to ensure equivalent accuracy across all conditions. In this process we identified, in the Zahn et al. materials, 11 social stimuli trials and 7 animal-function stimuli that had atypically low accuracy and determined that this was due to ambiguity in the response options of the original materials (e.g., both the target and foil were associated with the probe). In these cases, the unrelated foil word was replaced. Subsequently, equal accuracy was confirmed across conditions (all greater than 85% mean accuracy).

The psycholinguistic properties for the probes and choice words are provided in Table 2. There was no significant difference between the social and matched-abstract trials in imageability or semantic diversity (probes and choice words). The social and matched-abstract probes were matched for log frequency, though the choice words had a higher mean log frequency in the social condition (*t* = 2.6, *p* = 0.012). Both social probes (*t* = 3.1) and social choice words (*t* = 5.6) were significantly longer than those in the matched-abstract trials (both, *p* < 0.005). Independent sample *t*-tests confirmed that imageability ratings were higher for animal function trials compared with both social trials (probe *t* = 7.8; choice words *t* = 12.5) and matched-abstract trials (probe *t* = 7.8; choice word *t *= 10.3; all *p* < 0.05). There was no statistical difference in the log frequency of the animal function trials when compared with the social or the matched-abstract concepts. Words in the social concept trials were significantly longer than those in the animal function concept trials (both probes and choice words *t* > 3, *p* < 0.005), but there were no differences in word length between the matched-abstract trials and the animal function trials.

**Table 2:** Mean psycholinguistic properties of stimuli (range in parentheses)

Property	Social concepts		Matched-abstract concepts		Animal function concepts	
	Probes	Choices	Probes	Choices	Probes	Choices
Imageability	409 (270–587)	406 (285–587)	410 (270–571)	400 (290–553)	533 (411–638)	536 (344–632)
Semantic diversity	1.79 (1.19–2.06)	1.83 (1.37–2.18)	1.79 (4.57–2.14)	1.84 (1.47–2.16)	1.64 (0.99–2.14)	1.62 (0.99–2.32)
Log frequency	1.13 (0.16–2.18)	1.45 (0.32–2.65)	1.20 (0.38–1.98)	1.22 (0.25–1.99)	1.34 (0.02–2.69)	1.36 (0.41–2.71)
Length	7.6 (4–14)	8.2 (3–13)	6.2 (3–11)	6.1 (3–13)	5.8 (3–10)	5.5 (3–10)

Log frequency = log-transformed lemma frequencies from the CELEX database (Baayen et al. 1993). Imageability ratings were obtained from the MRC database (Coltheart 1981). Semantic diversity values obtained from Hoffman et al. (2013). Length = number of letters.

### Procedure

A PC running E-prime software (Psychology Software Tools, Pittsburgh, PA) was used for presentation of the stimuli and recording of responses. A block design was used, with each block lasting 13.5 s and consisting of three trials from the same experimental condition. Each trial began with a fixation cross presented in the center of the screen for 500 ms, followed by the stimuli (probe and choice words, simultaneously) in a black, lower-case font on a white background. Participants responded by pressing one of two designated buttons on an MR-compatible response box. The stimuli remained on the screen for a fixed duration of 4000 ms, at which point the next trial began.

The scanning procedure consisted of two runs or sessions of equal length (15 min) separated by a ten-minute interval. A single run contained 16 blocks of number judgment and 16 blocks of each of three semantic judgment conditions, all presented in a pseudo-random order. The procedure included three other semantic conditions which are the subject of separate hypotheses and analysis which are not reported here. The numerical judgment task also consisted of two conditions varying in difficulty. This manipulation was not relevant to this specific investigation and therefore, in the analyses reported below, these conditions were collapsed and treated as one.

### Imaging Acquisition

A key aim of the study was to assess whether processing of both social concepts and non-social general concepts elicits activation in the ventral anterior temporal lobe. It is therefore important to note that the conventional gradient-echo echo-planar imaging (EPI) technique employed for blood-oxygen-level dependent (BOLD) contrast is not equally sensitive to signal from different parts of the ATL; imaging of the ventral and polar ATL is particularly vulnerable to magnetic susceptibility artefacts that result in signal drop-out and geometric distortion in the phase-encode direction (Devlin et al. 2000; Visser et al. 2010a). Following our prior fMRI investigations, we reduced this problem by employing a spin-echo EPI imaging sequence, which greatly improves signal quality, combined with a post-acquisition k-space spatial correction for unwarping distortions (Embleton et al. 2010). We have previously demonstrated robust vATL activations for semantic tasks using this technique (Binney et al. 2010; Visser et al. 2010a; Visser and Lambon Ralph 2011; Visser et al. 2012; Robson et al. 2014; Binney and Lambon Ralph 2015; Hoffman et al. 2015a, 2015b). All imaging was performed on a 3 T Philips Achieva scanner using an 8 element SENSE head coil with a sense factor of 2.5. The spin-echo EPI fMRI sequence included 31 slices covering the whole brain with echo time (TE) = 70 ms, time to repetition (TR) = 3200 ms, flip angle = 90°, 96 × 96 matrix, reconstructed resolution 2.5 × 2.5 mm, and slice thickness 4.0 mm. 550 images were acquired in total, collected in two runs of 15 min each. Following the method of Embleton and colleagues (2010) for distortion-corrected spin-echo fMRI, the images were acquired with a single direction k space traversal in the left-right phase encoding direction. In between the two functional runs, a brief “pre-scan” was acquired, consisting of 10 volumes of dual direction k space traversal SE EPI scans. This gave 10 pairs of images matching the functional time series but with opposing direction distortions (10 left-right and 10 right-left). These scans were used in the distortion correction procedure (see below). A high resolution T2 weighted turbo spin echo scan with in-plane resolution of 0.94 mm and slice thickness 2.1 mm was obtained as a structural reference to provide a qualitative indication of distortion correction accuracy. In addition, a high resolution T1-weighted 3D turbo field echo inversion recovery image was acquired (TR ≈ 2000 ms, TE = 3.9 ms, Inversion time (TI) = 1150 ms, flip angle 8°, 256 × 205 matrix reconstructed to 256 × 256, reconstructed resolution 0.938 × 0.938 mm, and slice thickness of 0.9mm, SENSE factor = 2.5), with 170 slices covering the whole brain. This image was used for estimating transforms to warp functional images into standard stereotactic space (see below).

### Distortion Correction

The spatial remapping correction was computed using a method reported in detail elsewhere (Embleton et al. 2010). Briefly, in the first step, each volume from the functional time-series was registered to the mean of the distorted pre-scan images using SPM8’s (Statistical Parametric Mapping software; Wellcome Trust Centre for Neuroimaging, London, UK) 6-parameter rigid-body registration algorithm with 2nd degree B-spline interpolation. Although this initial step was taken primarily as part of the distortion correction procedure, it also functioned to correct the functional EPI volumes for minor motion artefacts. Subsequently, a spatial transformation matrix was calculated from the opposingly-distorted pre-scan images that consisted of the spatial-remapping necessary to correct geometric distortion. This transformation was then applied to each of the co-registered functional volumes resulting in two distortion-corrected datasets (one per run) of 225 volumes maintaining the original temporal spacing and TR of 3200 ms.

### fMRI Data Analysis

All of the following pre-processing steps and analyses were carried out using SPM8. The T1-weighted structural scan of each participant was first co-registered to a mean of their motion and distortion-corrected images (see above) using a six parameter rigid-body transform and the normalized mutual information objective function. SPM8’s unified segmentation and the DARTEL (diffeomorphic anatomical registration though an exponentiated lie algebra; Ashburner, 2007) toolbox were used to estimate a spatial transform to register the structural image to Montreal Neurological Institute (MNI) standard stereotaxic space. This transform was subsequently applied to the co-registered functional volumes which were resampled to a 3 × 3 × 3 mm voxel size and smoothed with an 8mm full- width half-maximum Gaussian filter.

Statistical analysis of the data employed the general linear model approach with a restricted maximum likelihood estimation. At the within-subjects level, each imaging run was subject to a separate fixed effect analysis. Each of the three semantic conditions and two numerical conditions was entered as a separate regressor; the blocks were modeled with a boxcar function and subsequently convolved with the canonical hemodynamic response function. Motion parameters were also entered into the model as covariates of no interest. Data were treated with a high-pass filter with a cut-off of 128 s. Contrast images were calculated to assess differences in activations between each of the semantic conditions and the control task (social concepts – numbers, matched-abstract concepts – numbers, and animal function concepts – numbers). Subsequent multi-subject analyses were carried out with second-level random effects analyses directed at our a priori hypotheses, as follows.

First, to examine areas activated by semantic processing in general (i.e., activation common to all conditions), we combined the contrasts of each semantic condition against numbers within a one-way ANOVA model (assuming dependence and equal variance) and by performing a *t*-test on the contrast [1 1 1] vector. The resulting statistical map was assessed for cluster-wise significance using a cluster-defining threshold of *p* < 0.001, uncorrected, and a false discovery rate-corrected cluster extent threshold at *p* < 0.05 (106 voxels) to control for the multiple comparisons problem (calculated per SPM8 under the random field theory (RFT) framework; search volume = 59 200 voxels; estimated smoothness [Full-width Half-maximum (FWHM) in mm] = 14.6, 14.3, 10.1). This global contrast can reflect common processing but can also just reflect strong activation in only a subset of the conditions (Nichols et al. 2005). Therefore, an additional conjunction analysis was performed which only reveals voxels that are activated for all 3 of the semantic conditions (Nichols et al. 2005). Here we used an uncorrected voxel-height threshold of *p* < 0.005 to be achieved by each contrast independently prior to conjunction.

**Figure 1.**
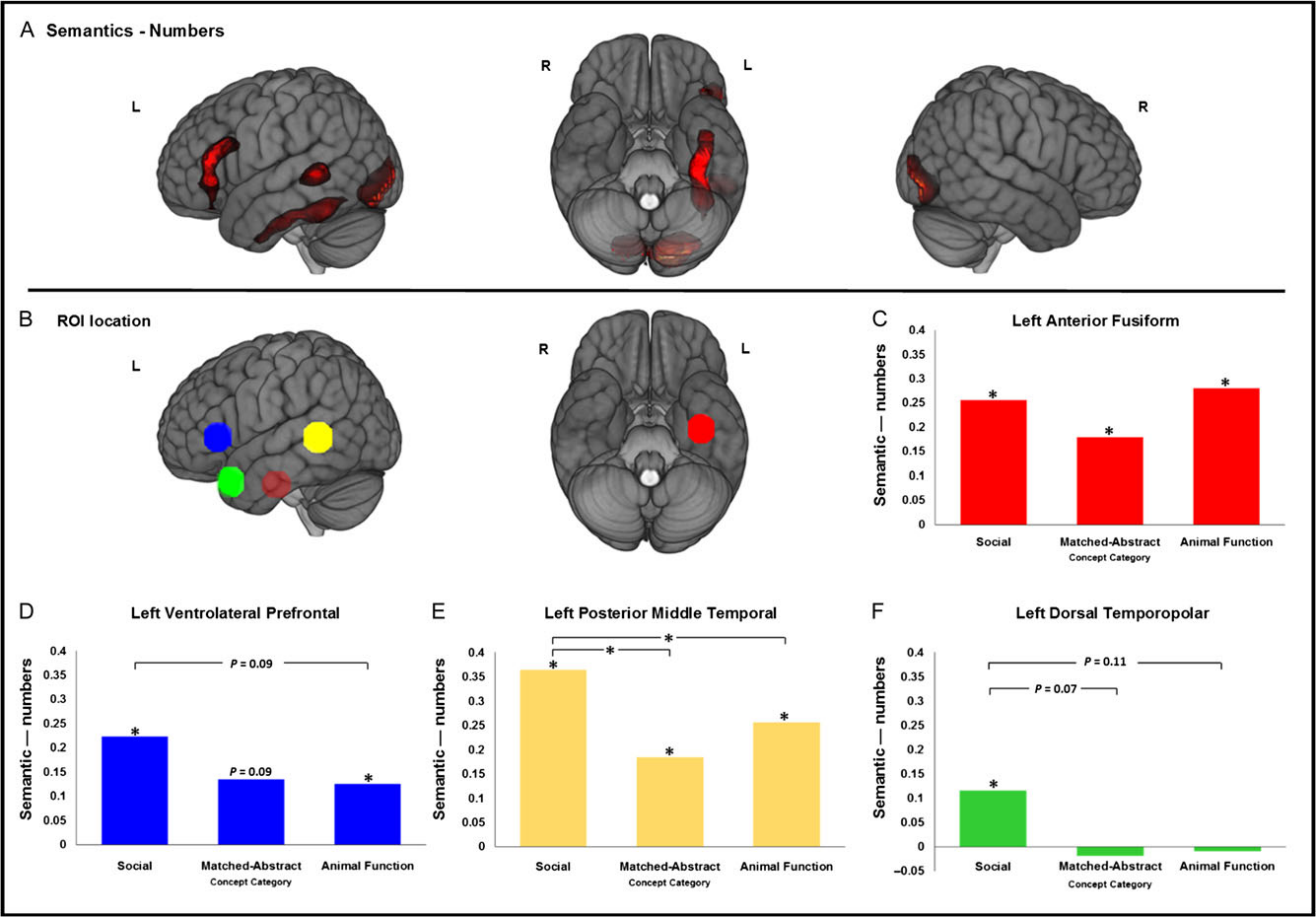
Cortical regions activated by the semantic judgment conditions relative to the number judgment condition. (*A*) Rendered results of the whole-brain analysis contrasting the combined semantic conditions with number judgment. The statistical map is thresholded with a voxel-height threshold of *p* < 0.001, uncorrected and a false discovery rate-corrected minimum cluster extent threshold at *P* < 0.05. Overlay brightness indicates distance from cerebral surface. (*B*) Locations of the regions of interest (ROIs). (*C–F*) Summary of the ROI analysis comparing the semantic activation (relative to number judgment) for each of three concept types (social, animal function and matched-abstract non-social concepts) across four components of the cortical semantic network. An asterisk denotes a significant effect at *p* < 0.05 after Bonferroni correction. *p*-values are displayed where effects were associated with a Bonferroni-corrected p-value of less than 0.15. R = Right hemisphere; L = Left hemisphere.

A key aim for this study was to assess directly whether the components of the core semantic network including ATL are equivalently recruited for processing of both general (non-social) and social concepts. To this end, we examined semantic activation within three a priori regions of interest (ROIs) using the MarsBar toolbox (Brett et al. 2002). These ROIs corresponded to three left-hemisphere regions commonly activated in functional imaging studies of semantic cognition and language (Binder et al. 2009; Visser et al. 2010b). Their precise locations were defined on the basis of results obtained from an independent dataset previously reported in Binney et al. (2010), where we used similar semantic judgment (which did not probe social concepts) and numerical judgment tasks. Peak activation coordinates defined a center of mass for three spheres with a radius of 10 mm (volume = 3984 mm^3^). These ROIs included the left ventral ATL (vATL; centered on the anterior fusiform gyrus [MNI coordinates = −36, −15, −30]), the left ventrolateral prefrontal cortex (vlPFC; centered on the pars triangularis [−54, 24, 3]) and the left posterolateral temporal cortex (encompassing the ventral superior and dorsal middle temporal gyri and the superior temporal sulcus [−66, −42, 3]; see Figure 1, Panel *B* for an illustration of ROI locations). In addition, we included a fourth ROI over the left dorsal temporopolar cortex, given that prior studies have implicated this ATL sub-region in social processing (Zahn et al. 2007; Ross and Olsen 2010). The coordinates [MNI: −51 16 −27; converted from the reported Tailarach −48 16 −20, using the tal2icbm_spm.m function – (http://biad02.uthscsa.edu/icbm2tal/)] to define this ROI's location were taken from the study of Ross and Olsen (2010) where left temporopolar activity for social versus animal function concepts was demonstrated in a prior replication of the Zahn et al. (2007) study. Per subject, a single summary value was calculated to represent activation across all voxels in a given ROI (median of the parameter estimates) for each semantic condition, relative to the number baseline. One-sample *t*-tests were then performed to assess group-level significance. To control for multiple comparisons, *p*-values were Bonferroni corrected on the basis of the number of ROIs (multiplied by 3). Within each ROI, we then compared activation between semantic conditions using paired *t*-tests (also Bonferroni corrected).

The next analyses examined the main hypothesis set out in the Introduction concerning the contributions of different ATL subregions to social and non-social abstract concepts. Next, we contrasted activation for the social and animal function concept trials in a whole-brain analysis using a paired *t*-test, [(social concepts – numbers) – (animal function concepts – numbers)]. Then, in a further whole-brain analysis (paired *t*-test), we contrasted the social concept condition with our new psycholinguistically-matched abstract (non-social) condition [(social concepts – numbers) – (matched-abstract concepts – numbers)]. To enable direct comparisons between the results of these contrasts in the present study and that of Zahn et al. (2007), we (a) display the statistical maps at the same statistical threshold used in their figures (an uncorrected voxel-height threshold of *p* < 0.005 and a minimum cluster extent threshold of 10 voxels) and (b) performed inferences at the same two further thresholds used in their whole-brain analyses. First, maintaining a voxel-height threshold of *p* < 0.005 uncorrected, we assessed significance using a minimum volume/extent threshold at *p* < 0.05 with false discovery rate (FDR) correction across the whole brain. Second, we applied a more conservative family-wise error (FWE) corrected cluster extent threshold at *p* < 0.05 to further assess strength of activations (see Results for minimum number of voxels per cluster). The search volume for the comparison between social and animal function concepts was 59 986 voxels with an estimated smoothness of 14.8, 14.5, 10.2 FWHM in mm with 663 RESELS for FWE-correction under the RFT framework in SPM8. The search volume for the comparison between social and matched-abstract concepts was 59200 voxels with an estimated smoothness of 14.8, 14.6, 10.1 FWHM in mm (656 RESELS). Following Zahn et al. (2007), we also employed a small volume correction (SVC) within a bilateral ATL volume. SVCs control for Type I errors and when, using voxel-level inferences, increase localization power compared with cluster based inferences. A FWE-corrected voxel-height threshold of *p* < 0.05 was applied to control for Type II errors. This bilateral ATL volume was defined on the basis of a map of temporal lobe hypometabolism reported in Nestor et al.’s (2006) prior (18 F) fluor-2-deoxy-D-Glucose positron emission tomography (FDG-PET) study of semantic dementia, as described by Binney et al. (2010). The left and right “mirrored” ATL ROIs we previously described were combined into a single bilateral ROI with a volume of 2760 voxels.

Finally, we implemented within-subjects models with the exact same variables as above, but with the addition of response times as additional regressors (obtained from pilot behavioral testing). This was to test whether any between-condition effects could be due to task difficulty. Due to the block design employed, there was a single value for each epoch of a condition which was the average of response times across the three trials within that block.

## Results

### Behavioral Data

Mean accuracy and response times in each condition are shown in Table 3. Differences between conditions were assessed using paired-sample *t*-tests. Overall, performance on the number baseline task was comparable to the semantic task, particularly the matched-abstract condition (accuracy *t* = 1.37, *p* = 0.19; response time *t* = 1.9, *p* = 0.07). This confirms that the number task was a suitable baseline for controlling effects of working memory, attention and executive skills associated with general cognitive processing. Whilst the numerical differences were small, statistically speaking, accuracy was lower for the social concepts (*t *= 2.35, *p* = 0.03) and animal function concepts (*t* = 2.8, *p* = 0.01) compared with the numerical baseline. There were no significant differences in accuracy between any of the semantic conditions, although there was a near-significant lower accuracy for the social concepts compared with matched-abstract concepts (*t* = 1.97, *p* = 0.06). Analysis of response times revealed no statistical difference between the matched-abstract condition and the numerical baseline task, but decision times were slower for social (*t* = 10.1) and animal function concept (*t* = 4.3) conditions relative to numerical judgments (both *p* < 0.001). Social concepts were processed more slowly than the matched-abstract concepts (*t* = 12.2) and animal function concepts (*t* = 7.4; both *p* < 0.001). Response times were also significantly longer in the animal function condition compared with the matched-abstract concept condition (*t* = 3.1, *p* = 0.006). Overall, the social concept and animal function stimuli from the Zahn et al. (2007) study were more difficult than the novel non-social condition yielding slower response times, although accuracy was comparable.

**Table 3:** Behavioral data

Condition	% Accuracy	Response time (ms)
Social concepts	89.8 (8.9)	1823 (186)
Matched-abstract concepts	92.5 (7.1)	1520 (131)
Animal function concepts	90.9 (6.7)	1610 (187)
Number baseline	94.6 (3.1)	1456 (141)

Standard deviations in parentheses.

### General Semantic Activation and Activation Common to the Processing of Social and Non-social Concepts

A whole-brain analysis contrasting the combination of all semantics conditions with the numerical task revealed activation of a network of three left-hemisphere frontal and temporal regions that feature prominently in contemporary models of semantic cognition. We have also reliably observed these regions in a number of prior fMRI studies using numerous different semantic and control tasks (see Fig. 1, Panel *A*; MNI coordinates are reported in Table 4). This included a ventrolateral prefrontal cluster that arched over much of the length of the inferior frontal gyrus, reaching from pars orbitalis, over pars triangularis and up to the dorsal posterior IFG. A posterolateral temporal cluster included the middle temporal gyrus but peaked in the superior temporal sulcus. In line with our previous distortion-corrected fMRI studies, there was a large cluster of ventral temporal activation encompassing much of the length of the fusiform gyrus and the anterior inferior temporal gyrus. This began in the posterior fusiform at *y* ≈ −63, extending anteriorly along the anterior fusiform to the anterior inferior temporal gyrus/sulcus at the position adjacent to the posterior border of the temporal polar cortex (Brodmann's are (BA) 38; *y* ≈ −0). A large cluster of bilateral occipital activation was also observed, perhaps reflecting the greater visual complexity of orthographic stimuli relative to digit stimuli, or semantic feedback to early visual areas (Hon et al. 2009). In addition, activation was observed in the right posterior lobe of the cerebellum.

**Table 4:** Significant activation clusters in the All-Semantics minus Numbers contrast (*p *< 0.05, FDR-corrected; with a cluster defining threshold of *p* < 0.001, uncorrected)

Cluster name (and peak locations)	Cluster extent (voxels)	Peak Z	Maxima MNI coordinates		
			*x*	*y*	*z*
L posterolateral temporal lobe	158	5.56	−54	−39	3
L ventral temporal lobe	248				
Posterior fusiform		5.25	−39	−48	−21
Anterior fusiform		4.84	−36	−21	−30
Anterior ITG/ITS		3.57	−39	−3	−42
L ventrolateral prefrontal cortex	221				
Pars triangularis		4.48	−51	30	12
Posterior dorsal IFG		4.08	−45	21	21
Pars orbitalis		4.07	−51	30	0
Bilateral occipital Lobe	679				
L lingual gyrus		8.07	−15	−90	−12
R lingual gyrus		6.43	18	−87	−6
R cerebellum	106	5.36	15	−78	−36

Table shows up to 3 local maxima per cluster that are more than 8.0 mm apart. L = left; R = right; ITG = inferior temporal gyrus; ITS = inferior temporal sulcus; IFG = inferior frontal gyrus.

A conjunction analysis revealed the same pattern of activation, suggesting that these regions form a core semantic network that supports processing to social and non-social alike. More specifically, this analysis yielded frontal and temporal activation clusters in the left posterior superior temporal sulcus (extent = 41 voxels; MNI coordinates = −57, −39, 3), the left posterior (22 voxels; −39, −45, −21) and anterior (16 voxels; −36, −18, −30) fusiform gyrus, and in pars triangularis of the left frontal operculum (12 voxels; −51, 30, 12). Clusters in the left (183 voxels; −15, −87, −12) and right (82 voxels; 18, −87, −6) occipital lobes and the right cerebellum (24 voxels; 15, −78, −30) also remained in this stringent analysis. The clusters were fractionated and considerably smaller relative to the map generated from the above global contrast. This may reflect the more stringent nature of the conjunction analysis and therefore a differential sensitivity to the same activation. Alternatively, the greater extent of activation in the global contrast may reflect greater activation in one of the conditions relative to the others. The following set of analyses examined these possibilities.

### Comparing Activation of the Core Semantic Network During the Processing of Social Versus Matched-abstract or Animal Function Concepts

We used an a priori ROI-based approach to compare regional responses to each of the three semantic conditions, focusing upon the left anterior fusiform gyrus, the left posterior MTG/STS, the left inferior frontal gyrus (IFG) and the left dorsal temporopolar cortex. The positions of these ROIs are illustrated in Figure 1, Panel *B*. The results of these analyses are displayed in Figure 1, panels *C*–*F*. Significant effects (*p* < 0.05, Bonferroni corrected) are denoted with an asterisk, and corrected *p*-values are displayed if less than 0.15. A central observation was that the left anterior fusiform was significantly activated (relative to numbers) in all of the semantic conditions, and that there were no significant differences between the conditions (Fig. 1, Panel *C*). An increasing body of research converges on this sub-region of the anterior temporal lobe as a core substrate for semantic memory (Binney et al. 2010; Mion et al. 2010; Peelen and Caramazza 2012; Shimotake et al. 2014; Abel et al. 2015). Our prior distortion-corrected fMRI studies have reliably demonstrated robust activation of this region to a wide range of semantic tasks in the verbal and non-verbal domains (Binney et al. 2010; Visser et al. 2010a; Visser and Lambon Ralph 2011; Visser et al. 2012; Hoffman et al. 2015a, 2015b) but it has been notably absent from fMRI studies of processing of social concepts (e.g., Zahn et al. 2007; Ross and Olsen 2010). By replicating the study of Zahn et al. (2007) in conjunction with distortion-corrected fMRI, we have demonstrated that these absences can, at least in part, be explained by the sensitivity of imaging protocols previously employed. As such this result constitutes unprecedented evidence to support the extension of the hypothesis regarding a role of this ventral anterior temporal region in semantic cognition to the processing of social concepts and the network sub-serving social cognition more generally.

The left posterior middle temporal ROI was significantly active in all semantic conditions (Fig. 1, Panel *E*) which is consistent with its purported role in semantic cognition. This activation, however, was significantly greater in the social concept condition than in the matched-abstract and the animal function conditions. As noted above, we observed longer response times for the social concept condition relative to the other semantic conditions and therefore these differences in activation may reflect differences in task difficulty between the conditions. This is consistent with a hypothesis, derived from patient studies, TMS and functional imaging studies, that this region subserves executive-semantic processes (Noonan et al. 2010; Whitney et al. 2011; Jefferies 2013). Given the longer average word-length in this condition, it could also reflect greater phonological complexity and associated processing demands. A similar pattern of activation was observed in the left IFG (Fig. 1, Panel *D*). Semantic activation was significant in the social condition and the animal function condition, and near significant in the matched-abstract condition (*p* = 0.09). There was a near-significant difference in the degree of activation between the social condition and the animal function condition (*p* = 0.09). It is well-established that this region is involved in retrieval, selection and regulation of semantic knowledge and activates more robustly when such task demands increase (Thompson-Schill et al. 1997; Badre and Wagner 2007; Hoffman et al. 2010, 2015a). This activation pattern may therefore also reflect task difficulty differences between the social and non-social conditions.

Finally, the left dorsal temporopolar ROI was only significantly active in the social concept condition (Fig. 1 Panel *F*) consistent with a putative role in social processing (Zahn et al. 2007; Ross and Olsen 2010). Only near-significant differences were observed between the response of this region to the social concepts and the other concept categories. Indeed, the response of this region to the social concept stimuli was small in comparison to the anterior fusiform and the other two perisylvian ROIs.

### The Lateral Anterior Temporal Lobe and the Processing of Social Concepts

**Figure 2.**
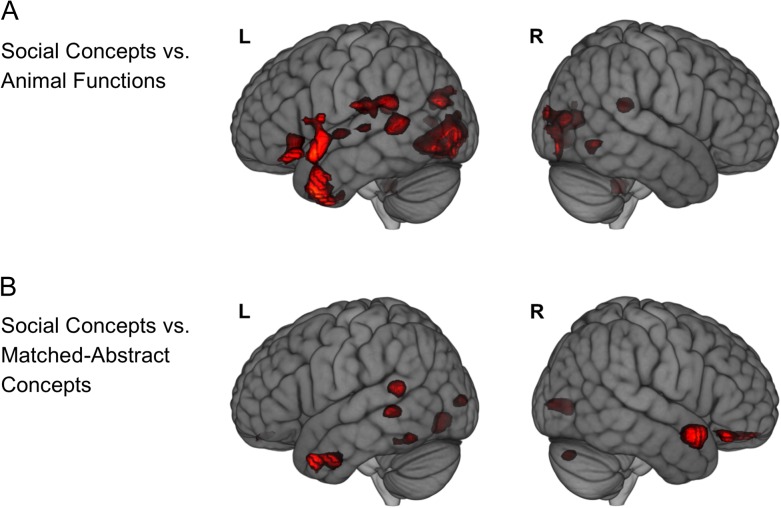
Whole-brain contrast of social concepts with non-social concept categories. (*A*) Replication of the contrast of social concept judgments and animal function concept judgments reported by Zahn et al. (2007). (*B*) Contrast of social concept judgments with judgments made on a novel set of stimuli matched to the former on lexical frequency, imageability and diversity of meaning. Both statistical maps are displayed with an uncorrected voxel-height threshold of *p* < 0.005 and a minimum cluster extent threshold of 10 contiguous voxels, as per Zahn et al. (2007). Overlay brightness indicates distance from cerebral surface.

Two separate research laboratories, using the same stimuli sets, reported superior anterior temporal lobe activation for the processing of social concepts relative to that of (non-social) animal function concepts (Zahn et al. 2007; Ross and Olsen 2010). We sought to replicate these findings by examining this same contrast in a whole-brain analysis. The resultant statistical parametric map is displayed in Figure 2, Panel *A*, at the same threshold used by Zahn and colleagues used in their figures (voxel height threshold of *p* < 0.05, uncorrected and a cluster extent threshold of 10 contiguous voxels). Clusters surviving the first threshold, a whole-brain false-discovery rate corrected extent threshold at *p* = 0.05 (121 voxels), are presented in Table 5, and only these clusters shall be discussed here. We observed activation at the left temporo-parietal junction (TPJ), including the supramarginal gyrus and (consistent with the ROI analysis) the posterior MTG, that extended into the posterior insular cortex. There were also two medial occipital clusters; a left hemisphere cluster at the superior aspect of the cuneus, and a bilateral cluster peaking at the lingual gyri. Of these three clusters, only the lingual gyri cluster survived the more-stringent whole-brain family-wise error corrected threshold (minimum cluster volume of 386 voxels). Zahn et al. (2007) reported similar temporo-parietal and medial occipital activations in their whole-brain analysis (significant after FDR correction). Why these regions are more robustly active for social concepts relative to animal function concepts is beyond the scope and objective of the current study, but it may reflect differences in word length and therefore processing demands associated with greater orthographic complexity (which were greater in the social stimuli; see Methods). More importantly, consistent with Zahn et al. (2007) and Ross and Olsen (2010), we observed a greater activation for social concepts relative to animal function concepts in the lateral anterior temporal lobe. There was a broad cluster (surviving both an FDR correction and a more stringent family-wise error corrected threshold at *p* < 0.05, as in the study of Zahn and colleagues) that encompassed the left basal, inferolateral and superior temporopolar cortex, extended posteriorly along the anterior superior temporal gyrus with caudal extent of ≈ −11, and dorsally to include the pars orbitalis of the inferior frontal gyrus (see Fig. 2, Panel *A* for locations). Following small volume correction restricting the analysis to the bilateral ATL (see Methods), voxel-wise inferences (*p* < 0.05, FWE-corrected; 25.4 RESELS) revealed a single significant peak in the left ventrolateral temporopolar cortex (−48, 9,−39; *z* = 5.0). Note that both of the prior studies reported activation of the anterior middle temporal gyrus, while Zahn and colleagues reported an additional, and more robust, activation of the superior temporal gyrus and superior temporal pole (BA38). In the present study, the lateral ATL cluster encompassed both of these regions.

**Table 5:** Significant activation clusters in the Social Concepts minus Animal Function Concepts whole-brain analysis (*p* < 0.05, FDR-corrected; with a cluster-defining threshold of *p* < 0.005, uncorrected)

Cluster name (and maxima locations)	Cluster *p*-value (FWE-corrected)	Cluster *p*-value (FDR-corrected)	Cluster extent (voxels)	Peak *Z*	Maxima MNI coordinates		
					*x*	*y*	*z*
L lateral ATL, vlPFC and insula	<0.001	<0.001	386				
Ventrolateral temporopolar cortex				5.00	−48	9	−39
Anterior STG				4.87	−57	9	−12
Basal temporopolar cortex				4.21	−39	3	−48
Bilateral occipital lobe	<0.001	<0.001	942				
L medial lingual gyrus				4.95	−15	−87	−9
R medial lingual gyrus				4.08	3	−84	−6
L lateral lingual gyrus				4.07	−33	−72	−9
L superior cuneus	0.116	0.038	121				
				4.31	−12	−78	24
				3.95	−27	−78	24
				3.32	−15	−87	30
L inferior lateral parietal lobe and insula	0.113	0.038	122				
Supramarginal gyrus				4.11	−54	−39	21
Posterior insular cortex				3.95	−48	−24	18
Opercular inferior parietal lobule				3.59	−60	−21	18

Table shows up to 3 local maxima per cluster that are more than 8.0 mm apart. L = left; R = Right; ATL = anterior temporal lobe; vlPFC = ventrolateral prefrontal cortex; STG = superior temporal gyrus.

An important difference between the results of the present study and those of these two previous investigations concerns the hemispheric laterality of activation for social concepts relative to animal function concepts. We observed lateral ATL activation only in the left hemisphere, even at a liberal statistical threshold (see Fig. 2, Panel *A*). Zahn et al. (2007) and Ross and Olsen (2010) both observed bilateral activation, with the former observing right greater than left (right > left) ATL activation and the latter reporting left greater than right (left > right) ATL activation. This calls into question any claims regarding the strength of ATL laterality for social conceptual processing. A potential contributor to such variability in laterality is uncontrolled/unmodelled variability in stimulus characteristics other than the categorical distinction of social and animal function concepts, particularly psycholinguistic variables commonly associated with semantic processing such as imageability and word frequency. Indeed, Zahn and colleagues included a number of these variables in their regression model as covariates of no interest, whereas this was not possible in the experimental design of Ross and Olsen. This difference in analytical procedures could offer a partial explanation for the laterality differences in the effect size of their observed lateral ATL activations. Indeed, in the present study, rather than partial out variables within a regression, we were able to contrast the social condition with a novel non-social condition that was closely matched on a number of such psycholinguistic variables, a priori. We observed an effect in both the right and left lateral ATL, as follows.

The statistical parametric map revealed by contrasting the social condition with the matched-abstract condition is displayed in Figure 2, Panel *B*, at the same threshold used in Zahn et al.’s (2007) figures. There were no significant effects in the whole-brain analysis at an FDR-corrected (or FWE-corrected) cluster volume threshold of p < 0.05, and therefore this comparison yielded much smaller differences in activation than the comparison with animal function concepts. Following small volume correction, voxel-wise inferences (*p* = 0.05, FWE-corrected; 26 RESELS) yielded significant activation in the right anterior STS/STG (57, 9, −18; *z* = 4.24) and another in the left anterior MTG/temporopolar cortex (−54, 9, −33; *z* = 4.04; see Fig. 2, Panel *B* for locations). The effect size in the left and right ATL were comparable in the present study. The right ATL activation was at a similar location to that reported by Zahn et al. (2007; whole-brain/ROI analysis right ATL peak coordinates = 57, 12, 0/51, 18, −12). Their left ATL activation was in the superior and middle temporal gyri. It is possible that they did not observe activation in the more inferior polar cortex due to magnetic susceptibility artefacts (which were alleviated by our imaging protocol).

Finally, we examined whether any of the above reported differential activation could be explained by task difficulty by including response times for each condition as additional regressors at the single-subject level and repeating the same exact group-level analyses. All of the observed effects remained significant, except that the greater activation for social relative to animal function concepts became limited to the left ATL and medial occipital regions. There was no direct association between response times and anterior temporal lobe activation.

## Discussion

Recent fMRI investigations have led some researchers to propose that the superior ATL is a domain-specific representational substrate for socially-relevant concepts (Zahn et al. 2007; Ross and Olsen 2010; Skipper et al. 2011). In parallel, the ventrolateral ATL (vATL) has emerged as a core “center-point” region for the representation of general conceptual knowledge (Binney et al. 2010; Mion et al. 2010; Lambon Ralph 2014; Shimotake et al. 2014). Taken together, these parallel results might suggest a functional division in the ATL. We investigated whether these two hypotheses could instead be reconciled within a single unified theory of semantic function that posits the ATL region as a graded transmodal representational hub (Visser and Lambon Ralph 2011; Binney et al. 2012; Visser et al. 2012; Shimotake et al. 2014; Abel et al. 2015; Rice et al. 2015a, 2015b). The three key empirical findings were as follows:

1. By utilizing distortion-corrected fMRI, active contrast conditions and a full field-of-view, we were able to confirm that the absence of the vATL in the previous studies of social concept processing reflects the limitations of conventional fMRI protocols in obtaining reliable signal from this region.
2. Social, animal-function and the new matched-abstract concepts commonly engaged a core left-hemisphere semantic network comprising the inferior frontal gyrus (IFG), the posterior middle temporal gyrus (pMTG) and the ventral ATL (vATL). The IFG and pMTG exhibited greater activation for the social concepts while the response of the vATL was equivalent for all three concept categories.
3. In addition, although weaker than in the “omni-category” vATL, social concepts generated relatively greater activations in the bilateral superior ATL (sATL). This graded difference remained even when social concepts were compared with a set of non-social concepts, tightly matched for various key semantic psycholinguistic variables, although the effect size was smaller. Accordingly, it would appear that the weaker yet differential activation for social concepts in sATL rules out any explanation purely in terms of semantic quantitative factors (e.g., semantic richness or diversity).

### The Graded ATL Semantic Hub

Two decades of detailed neuropsychological investigation of semantic dementia (SD) has led to the hypothesis that the (bilateral) ATL plays a role in the transmodal representation of conceptual knowledge (Rogers et al. 2004; Patterson et al. 2007; Lambon Ralph et al. 2010; Lambon Ralph 2014), bolstered by an accumulation of convergent evidence from both functional imaging (including PET, fMRI and MEG) and transcranial magnetic stimulation studies of healthy subjects (Vandenberghe et al. 1996; Marinkovic et al. 2003; Pobric et al. 2007; Lambon Ralph et al. 2009; Visser and Lambon Ralph 2011).

Research efforts have now begun to refine neuroanatomical hypotheses regarding the roles of this relatively large swathe of cortex (Binney et al. 2010). Recent distortion-corrected fMRI studies suggest that a critical region for conceptual processing lies in the anterior fusiform and inferior temporal gyrus (which we term here the ventrolateral ATL; Binney et al. 2010; Visser et al. 2010a). Strikingly, the vATL is one of the most atrophied ATL regions in SD (Galton et al. 2001) and hypometabolism of the anterior fusiform in particular correlates with multimodal semantic impairments in these patients (Butler et al. 2009; Mion et al. 2010). PET and distortion-corrected fMRI studies and a recent intracranial recording study, have confirmed the transmodal nature of the bilateral vATL by demonstrating equivalent responses to spoken words, written words, pictures and non-verbal sounds (Vandenberghe et al. 1996; Spitsyna et al. 2006; Visser et al. 2012; Shimotake et al. 2014; Abel et al. 2015). In addition, recent representational similarity analyses of fMRI and cortical grid data find direct evidence of semantic coding at this region (Peelen and Caramazza 2012; Coutanche and Thompson-Schill 2015; Chen et al. 2016).

Some researchers have proposed that the vATL is primarily involved in processing sensorimotor feature knowledge (Bonner et al. 2009) and therefore only serves in the representation of concrete concepts (Bonner and Price 2013). To the contrary, we have recently demonstrated that the vATL activates during semantic judgments on concrete and abstract words (Robson et al. 2014; Hoffman et al. 2015a). As such, it emerges as a substrate for concepts of all types. Here we have also demonstrated vATL engagement in processing social concepts. As far as we are aware, there is no precedent evidence for a role of the vATL in social cognition with prior conjecture concerning the ATL being limited to polar or superior aspects. Whether its role is limited to comprehension of words used to describe the social world or extends to processing of non-verbal social cues remains to be shown. Further distortion-corrected fMRI studies are required to explore vATL responses during non-verbal social tasks typically employed in the social neuroscience literature (e.g., theory of mind/social inference tasks; Ross and Olsen 2010).

### Differential Engagement of the superior ATL for Social Concepts

We replicated prior observations of a differential activation of the bilateral superior ATL (sATL) for social concepts relative to non-social concepts. Rather than consider this region as a selective, domain-specific “module” dedicated to representation of socially-relevant conceptual knowledge (Zahn et al. 2007; Ross and Olsen 2010; Skipper et al. 2011), an alternative explanation is required. Our results suggest that social concept processing is distributed over the bilateral ATL, including the vATL where the activation for social concepts is equivalent with that for other types of concept and the sATL where activation is only relatively greater for social concepts compared with non-social concepts (also see Rice et al. 2015a). Moreover, the response of the vATL to social concepts was greater than that of the sATL.

We hypothesize that the graded pattern of ATL involvement in social and non-social concepts follows from a connectivity-driven graded variation in semantic function across the ATL. It is increasingly apparent that there are graded differences both within and across the ATLs in terms of connectivity (Ding et al. 2009; Binney et al. 2012; Pascual et al. 2013; Hurley et al. 2015; Jackson et al. 2016) and fMRI activations (Visser and Lambon Ralph 2011; Visser et al. 2012; Rice et al. 2015b). Accordingly, we have recently proposed a model of the bilateral ATL as a graded transmodal representational substrate, with gradation of semantic function arising from differential connectivity and proximity to input sources (for a computational exploration of this general hypothesis, see Plaut 2002). Intra-ATL connectivity drives conjoint lateral and rostral convergence of information from multiple modalities, culminating at the hub's center-point in the vATL (Binney et al. 2012; Lambon Ralph 2014; Rice et al. 2015a). Away from this point, including towards the superior ATL, semantic function is nuanced by the greater influence of information from a given modality (Visser and Lambon Ralph 2011; Visser et al. 2012; Hoffman et al. 2015a). Given the strong connectivity to medial temporal limbic and frontal limbic regions (via the uncinate fasciculus; Binney et al. 2012; Bajada et al. 2016; Papinutto et al. 2016), the dorsal-polar ATL regions may become important for the assimilation of emotion and valence-related information into coherent semantic representations (Troche et al. 2014; Rice et al. 2015a). Our matched non-social abstract concepts, of course, do not contain these features and thus, this could contribute to explaining the social>non-social distinction observed in the dorsal-polar ATL area. In contrast, the maximal convergence of multiple sources of input into the vATL generates its omni-category characteristic and thus its equivalent and considerable contribution to social and non-social concepts.

Further support for this graded framework for a role of the ATL in social processing exists in histology and patient dissociations. Social processing has been linked with temporopolar regions because these areas are associated with “limbic”, agranular cortex. Both historical and contemporary cytoarchitectural studies, however, have found that agranular cortex is limited to the medial temporopolar region with a gradual transition through dysgranular cortex at the tip of the pole to granular areas on the lateral and inferior surfaces (Brodmann 1909; Ding et al. 2009). Thus there appears to be a shift from “limbic” cortices in the medial pole to neocortex in the sATL and beyond. Further, in the initial stages of behavioral-variant frontotemporal dementia (which is primarily characterzied by socio-behavioral and personality changes), atrophy typically extends from orbitofrontal regions to the temporal pole alone (rather than the entire ATL region) without generating the same degree of semantic impairment observed in SD patients (Perry and Hodges 2000). This suggests that a somewhat purer form of socio-affective processing in ATL cortex could be localized to agranular/dysgranular polar cortex while damage to posteriorly-adjacent granular ATL neocortex (especially the vATL) is necessary for conceptual knowledge impairments.

Future work can examine the functional role of the sATL activations further by using multi-voxel pattern-based fMRI analyses, a data-driven technique developed for extracting information content and representational structure (e.g., Huth et al. 2016). We observed two distinct loci of activation in the polar cortex and sATL (Fig. 2). Moreover the activations of each of these subregions varied as a function of whether social concepts were contrasted to animal function or the matched-abstract concepts. Using pattern-based analyses it may be possible to glean further insight into the nature of information these different regions process and how they differentially contribute to the three concept categories. For example, a recent pattern-analysis study examined the neural basis of perceived threat of animals (or “predacity”) and identified increasingly pronounced and specific responses along the right dorsal temporal lobe culminating in the anterior superior sulcus (Connolly et al. 2016). The authors note associations of this region with a role in cognitive evaluations of aggressiveness or trustworthiness of other humans and their intentions towards oneself. Thus, this function appears to be shared with evaluations of perceived threat of non-human animals. Interestingly, we observed that the right superior temporal gyrus/sulcus only differentially responded to social concepts when they were compared with the matched abstract concepts but not the animal function concepts. Of course the social concept stimuli included words such as “truthful” and animal functions included “poisonous”, whereas the matched-abstract words were devoid of any meaning that implied threat. Thus these findings are consistent and could suggest a role of the right sATL in the evaluation of the intention of others, not limited to other human beings.

### The Role of the Left Versus Right ATL in Processing Social Concepts

Neuroimaging and neurostimulation studies are consistent with semantic dementia in implicating the bilateral ATL in semantic cognition (Patterson et al. 2007; Lambon Ralph et al. 2009; Rice et al. 2015a, 2015b). Bilateral ATL activations have been observed in semantic tasks for words, sounds, spoken names and pictures (Sharp et al. 2004; Binney et al. 2010; Visser and Lambon Ralph 2011). A left hemispheric bias has been reported for ATL activations when tasks are performed with written words or require a spoken output (Marinkovic et al. 2003; Rice et al. 2015b) which may reflect the stronger connectivity to the left-biased speech production and reading systems (Lambon Ralph et al. 2001; Schapiro et al. 2013; Hurley et al. 2015). The laterality for social concepts is less clear. Typically, fMRI explorations of socially-related concepts report bilateral ATL activations. Some studies have found a right hemisphere bias (Zahn et al. 2007; Skipper et al. 2011) whilst others have identified the opposite (Ross and Olsen 2010). Formal meta-analyses that combine data from across the literature find little evidence of any asymmetry (Rice et al. 2015b). Clinically, social impairments are often more apparent in the presentation of patients with right hemisphere damage. Indeed the social impairments of semantic dementia patients with right-biased atrophy have been used to argue for a selective contribution of the right ATL to social concepts (Olson et al. 2007; Chan et al. 2009; Zahn et al. 2009; Skipper et al. 2011; Kumfor et al. 2016). When formally assessed, however, semantic dementia patients with left asymmetric-ATL atrophy also have behavioral changes (Chan et al. 2009; Kumfor et al. 2016) though these can be masked in their clinical presentation due to the patients’ language impairments including their profound naming difficulties (also see Binney et al. 2016). Indeed, whilst Kumfor et al. (2016) obtained a significant correlation between right ATL atrophy and the level of behavioral impairment, the correlation with the left ATL region was only marginally smaller. There are two potential solutions to this apparent ATL asymmetry in the patient's presentation. The first is that social concepts are supported bilaterally across the ATL but the right ATL comes to play a more critical role due to greater connectivity to other socially-related regions through the uncinate fasciculus. The laterality of the uncinate fasciculus, however, is currently unclear as, although one post-mortem study found this tract to be larger in the right than left hemisphere (Highley et al. 2002), recent diffusion-weighted imaging studies have failed to replicate this asymmetry (Kubicki et al. 2002; Hasan et al. 2009). A second potential explanation comes from a recent study which compared the effect of transcranial magnetic stimulation (TMS) to the left versus right sATL on semantic decisions to social versus non-social concepts (Pobric et al. 2016). TMS to either sATL region generated a significant slowing for the social concepts but only left sATL stimulation slowed decisions on the non-social concepts. Thus, the results of this TMS investigation appear to mirror the pattern observed in SD patients. If correct then these results suggest that verbal rather than social processing is asymmetrically supported in left sATL regions and that the verbal deficits which follow after left ATL damage can mask the patients’ concurrent social processing impairments.

### Beyond the ATL

In addition to the ATL activations, we also observed significant involvement of prefrontal and posterior temporal areas in both social and non-social semantic processing. This is consistent with neuropsychological, functional neuroimaging and neurostimulation studies which have converged upon a distributed transmodal network for semantic cognition comprising the IFG, the posterior MTG (pMTG), intraparietal sulcus (IPS) and the ATL (Lambon Ralph 2014). Recent studies have suggested that this multimodal network reflects two core functions in semantic cognition: (i) long-term representation of coherent concepts (supported by interaction of the ATL transmodal hub with distributed modality-specific sources of information; Rogers et al. 2004); and (ii) semantic “control” (Jefferies and Lambon Ralph 2006; Jefferies 2013). Semantic control refers to a set of executive and working-memory related processes that are engaged to manipulate semantic information in line with task- and context-specific requirements. The role of the IFG in semantic control is well established from functional neuroimaging, neuropsychological studies and more recently repetitive transcranial magnetic stimulation (Thompson-Schill et al. 1997; Jefferies and Lambon Ralph 2006; Badre and Wagner 2007; Whitney et al. 2011). Another series of multi-method studies have also recently elucidated a similar executive-semantic role for the pMTG (Noonan et al. 2010; Whitney et al. 2011; Noonan et al. 2013). fMRI demonstrates that the response of IFG and pMTG is modulated by task difficulty (Thompson-Schill et al. 1997; Noonan et al. 2013). Patients with lesions in these areas exhibit multimodal semantic impairments but, critically, their task performance varies as a function of item and task difficulty (Jefferies and Lambon Ralph 2006; Noonan et al. 2010; Rogers et al. 2015). This is in contrast to semantic dementia (SD) patients who exhibit progressive bilateral anterior temporal atrophy associated with a selective and gradual dissolution of conceptual knowledge. This is evident irrespective of the specific task or input and output modalities (Bozeat et al. 2000; Coccia et al. 2004; Goll et al. 2009; Piwnica-Worms et al. 2010).
